# Gender mainstreaming and gender-disaggregated data on migrant women: WEMov’s Open Letter

**DOI:** 10.12688/openreseurope.18103.1

**Published:** 2024-09-09

**Authors:** Stellamarina Donato, Marie Ruiz

**Affiliations:** 1 Libera Universita Maria Santissima Assunta, Rome, Lazio, Italy; 2 CORPUS, Universite de Picardie Jules Verne, Amiens, Hauts-de-France, France

**Keywords:** gender, migration, data, mainstreaming, policy

## Abstract

The European Union (EU) stands as a mosaic of diversity, enriched by a substantial migrant population, where women play a vital role. Migrant women face vulnerabilities, often disproportionate to their male counterparts. Yet, they also make significant economic and social contributions as entrepreneurs, professionals, caregivers, activists, and members of their communities. This open letter is a brief that argues that gender mainstreaming and the utilization of gender-disaggregated data are crucial tools for addressing gender disparities and ensuring effective policymaking.

## Disclaimer

The views expressed in this article are those of the author(s). Publication in Open Research Europe does not imply endorsement of the European Union’s Framework Programme.

## Introduction

Gender mainstreaming is the systematic integration of a gender perspective into all policies, ensuring that migration policies consider the specific needs and lived realities of women ( EIGE, 2024). Gender-disaggregated data, collected and analysed separately for men and women, reveals these disparities and empowers evidence-based policy decisions.

This open letter draws on the expertise of the COST Action Women on the Move (CA19112) to examine the current state of gender mainstreaming and disaggregated data collection for migrant women in the EU. It identifies key challenges, proposes recommendations, and emphasizes the need for collaborative efforts and a cross-country perspective. The letter concludes with recommendations for policymakers, urging them to prioritize the collection and analysis of gender-disaggregated data on migrant women at the right level of granularity, encompassing various aspects like employment, healthcare, education, and their contributions to the local economies, while integrate a gender perspective into all stages of policy development, ensuring women's needs and challenges are addressed. The brief furthermore concludes the outmost importance of allocating resources for capacity building to raise awareness of gender-specific issues and equip policymakers with the necessary skills to utilize gender mainstreaming and disaggregated data effectively.

Through prioritizing gender-disaggregated data collection and implementing gender mainstreaming across EU policies, the EU can create a framework to achieve gender equality in migration and beyond, which will foster more inclusive and equitable societies and economies for all.

### I. Challenges identified by WEMov

This open letter draws upon the current research questions and issues:

Data Collection Challenges:

1.   What challenges exist in collecting consistent and reliable gender-disaggregated data on women’s migration across European countries, and how do privacy concerns impact data sharing among EU member states?

2.   In what ways do variations in data collection methodologies hinder the development of a standardized approach to gender-disaggregated data on women’s migration, and how can the EU promote such standardization? What would be the benefits of standardized gender disaggregated data at EU level? Who would benefit from gender-disaggregated data?

3.   Which EU countries do not yet include gender-disaggregated data? Why?

4.   A key challenge lies in collecting gender-disaggregated data at a granular level, such as the neighbourhood level, which is crucial for designing targeted policies and accurately analysing the socio-economic impact of migrant women on local communities.

5.   The importance of distinguishing between sex and gender-disaggregated data as to include gender-disaggregated data that overcome the non-binary classification of gender

Policy Implementation and Gender Mainstreaming:

6.   What are the key challenges in implementing gender mainstreaming practices in migration and linked policies, including economic, health and social across different European countries, and how can the EU enhance collaboration for better gender integration in women’s migration policies?

7.   How effective are existing legal frameworks, such as the TFEU and the Gender Equality Strategy 2020–2025, in promoting gender-disaggregated data collection and gender mainstreaming in migration policies, and what mechanisms can ensure compliance across EU member states?

Knowledge Transfer:

8.   What initiatives can be introduced to enhance the capacity of EU member states in collecting, analyzing, and utilizing gender-disaggregated data on women’s migration, and how can the EU facilitate knowledge transfer and best practice sharing for improved gender mainstreaming efforts?

9.   What use can we make of gender disaggregated data, in what context should they be used and how gender-disaggregated data can inform targeted interventions for specific migrant communities? What’s the role of the media in this context? And civil society organizations? Should gender-disaggregated data be used to nuance macro-discourses on migration?

These questions and issues provide a framework for examining the complexities and challenges associated with gender-disaggregated data and gender mainstreaming in the context of women’s migration in Europe, emphasizing the need for collaborative efforts and a cross-country perspective.

### II. General framework

According to the latest statistics from the EU Commission in 2021, the European Union is home to 446.7 million people, with 23.8 million being non-EU citizens, constituting 5.3% of the total population. Additionally, as of mid-2020, women slightly outnumbered men among international migrants in Europe, accounting for 51.6% of the total ( UN DESA, 2020). Hence, the representation of women in international migration is similar to that of men, yet conventional migration scholarship has largely overlooked their presence ( Boyd & Grieco (2003). This oversight is incongruent with the actual dynamics of international migration, thereby constraining our comprehension of its gender dimension ( Bircan & Yilmaz, 2023). One significant contributing factor to migrant women’s invisibility is the prevalence of gender-blind approaches within migration theories and data collection practices ( Hennebry & Petrozziello, 2019). These approaches neglect the unique realities experienced by male and female migrants, resulting in a restricted understanding of how gender intricately influences the entire migration process from pre-departure decisions to arrival, integration and thriving within local EU economies and societies and possible return.

Compounding this issue is the inadequacy and inconsistency evident in available quantitative migration data, but also in data sets related to key areas impacted by migration, such as labour markets, healthcare systems, and education systems. These descriptive statistic data suffer from notable limitations, such as insufficient information on the motives driving migration, incomplete coverage of various geographical regions and migrant demographics, and a failure to adequately address the gender dimension. For instance:

1.   Motives for migration such as economic opportunities, education, family reunification, or escaping gender-based violence, are often lacking. Understanding these motivations is crucial for formulating targeted policies that address the diverse needs of women migrants;

2.   Intersectionality, meaning the interconnected forms of discrimination and privilege that shape women’s migration experiences;

3.   Socio-economic impacts such as their contributions to the labour market, entrepreneurship, and overall societal development, as well as any challenges they face in accessing opportunities and resources in the host and origin countries;

4.   Unique health challenges faced by migrant women, such as maternal healthcare and reproductive rights;

5.   Legal and policy barriers such as gender equality in migration policies and gender gaps and discrimination in the host and origin countries;

6.   Violence and exploitation. These shortcomings make it difficult to unravel and analyse the intricate role of gender in international migration data ( Benería *et al.*, 2012).

As a matter of fact, some gender-disaggregated data is available at UN level, World Bank, OECD, IOM and EU levels but the methodology to collect data is scattered. For instance, the World Bank’s migration and remittances data ( https://www.worldbank.org/en/topic), lack a gender-specific breakdown, providing limited insight into the comparative data of international remittances sent by migrant females and males and hence undermining the role of migrant women in improving the economic development and living conditions of people in migrants’ countries of origin ( Guallar Ariño, 2023). And, the OECD International Migration Outlooks routinely release comprehensive statistics on total immigration and emigration flows for member countries, without providing a breakdown by gender ( OECD, 2023). Eurostat, instead, offers gender-specific totals for various European countries within their bilateral migration flow datasets. However, the data from or to each country may not align with a uniform definition of migration flow, as reporting practices vary based on national government needs and the design of migration statistical systems. Moreover, only Immigration by country of previous residence and emigration by country of next residence from 1989 to 2019 is the available Eurostat Data sources on total migration flows by sex ( Abel & Cohen, 2022).

Furthermore, the neglect of intersectionality, meaning the interconnected and cumulative impact of various forms of discrimination that significantly influence the everyday experiences of individuals, with a particular emphasis on women, and especially women of colour ( Crenshaw, 1991) in existing theories and data exacerbates the problem. Overlooking the interconnected factors influencing migration and the diverse nature of migrant groups creates a knowledge gap, impeding the development of gender-responsive migration governance and hindering the formulation of policies tailored to the specific needs and experiences of diverse migrant populations ( Ahmad-Yar & Bircan, 2021). Moreover, the skill-mining not only guarantees a more effective reception and integration process but also accentuates the valuable contributions of women migrants to receiving nations, thereby introducing nuance to and dispelling xenophobic fears surrounding migration. Henceforth, addressing these gaps is crucial to develop comprehensive and inclusive migration policies that consider the diverse experiences of both male and female migrants as well as people who identify with other genders.

With reference to the legal framework, the EU has shown commitment to gender equality through various legal frameworks, also abiding by the 2030 Agenda SDG, for example at target 17.18 which calls to increase the availability of “high-quality, timely and reliable data disaggregated by income, gender, age, race, ethnicity and migratory status”, including the Treaty on the Functioning of the European Union (TFEU) and the Gender Equality Strategy 2020–2025. The EU also monitors the situation per country thanks to the EIGE (European Institute for Gender Equality). EIGE uses gender mainstreaming, intended as the application of “a gender equality perspective in each phase of the policy-making cycle as well as all areas within policies and processes such as procurement or budgeting.”

Specifically for migrant women it remains clear that collecting gender disaggregated data is still a non-shared EU practice, for instance among EU Mediterranean countries that face migrants arrival by sea, until 2024 Spain’s official data on sex ratio is not available ( Guallar Ariño, 2023) whereas it is in Italy and Malta, as well as mainstreaming gender in policy practices on the topic.

Finally, the landscape of gender-disaggregated data and gender mainstreaming in women’s migration policies within the European Union (EU) is continually evolving. This state-of-the-art analysis explores the current challenges, advancements, and future prospects in understanding and addressing the complex intersection of gender, migration, and policy across European countries.

### III. Data collection

Despite the EU’s commitment to gender equality, challenges persist in collecting consistent and reliable gender-disaggregated data on women’s migration ( Decataldo & Ruspini, 2016). Privacy concerns and variations in methodologies hinder the development of a standardized approach across member states. This is conspicuous in countries like France that do not harmonize gender-disaggregated data and mention the countries of origin for return migration. Moreover, Germany and the UK, both hosting a considerable number of migrants, either fail to collect or omit the reporting of sex-disaggregated data to Eurostat. Additionally, the inconsistent presence of sex-disaggregated data for residence permits poses a challenge, notably for Croatia during 2009–2012, the Czech Republic, France, and Switzerland in 2010–2011, the Netherlands in 2010 and 2013–2015, and Luxembourg in 2010 and 2012–2013 ( Bircan & Yilmaz, 2023). This discrepancy can be attributed to the diverse reporting preferences of these countries, which emphasizes that it is unrealistic to assume the non-existence of sex-disaggregated data for residence permits across all EU member states. Consequently, this poses a significant impediment to the availability and accessibility of cross-country data, particularly for comparative research endeavours. As based on WEMov’s experience, we believe that addressing these challenges is essential to build a comprehensive understanding of migrant women’s experiences.

### IV. Policy implementation and gender mainstreaming

Progress has been made in integrating gender mainstreaming into migration policies, but implementation remains uneven across EU member states. There is a need for a cohesive approach to recognize and address the diverse experiences and vulnerabilities of migrant women and their impact on local economies the societies/communities.

Moreover, gender statistics are vital for social research and policy development, as they document the diverse influences of social norms and institutions on the experiences of both migrant women and men, resulting in gender inequalities. The collection of gender-sensitive statistical information is essential, driven by a growing demand from various sources such as society, institutions, and official agencies ( Decataldo & Ruspini, 2016).

As for specific laws and policy measures, the TFEU and the Gender Equality Strategy 2020–2025 serve as foundational legal frameworks for promoting gender equality, but their impact on the consistent collection of gender-disaggregated data needs further examination. Ensuring compliance and enforcement of gender mainstreaming practices across member states remains a critical area of focus.

In detail, the EU Gender Equality Strategy 2020–2025 emphasizes the need for gender mainstreaming in all EU policies, including migration. This strategy encourages member states to collect and utilize gender-disaggregated data to address the specific needs of migrant women. However, enforcement and standardization of gender mainstreaming practices are areas that require further attention.

The EU Asylum and Migration Pact, though primarily focused on asylum and migration issues, has the potential to incorporate a gender-sensitive approach. It calls for the collection of data on the vulnerability of asylum seekers, which should include gender-disaggregated data. This is a step in the right direction but needs to be consistently implemented across the EU. Finally, the EU Anti-Trafficking Directive recognizes the gender-specific aspects of human trafficking and mandates the protection of victims, many of whom are migrant women. This directive underscores the importance of gender mainstreaming in policies related to human trafficking but should be extended to other migration-related areas.

These instruments emphasize the importance of integrating gender perspectives into all areas of policy-making throughout the complete policy cycle. However, implementation remains uneven across member states and sectors.

### V. Knowledge transfer

Initiatives to enhance the capacity of member states in collecting and utilizing gender-disaggregated data are essential. Knowledge transfer mechanisms should be implemented to share best practices, fostering a collective effort to improve gender mainstreaming in migration policies.

Establishing a robust monitoring and evaluation framework is crucial for assessing the effectiveness of gender-disaggregated data collection and gender mainstreaming initiatives. Identifying indicators and benchmarks for progress is imperative for guiding future policy development.

Collaboration among governmental agencies (i.e. national statistical offices), non-governmental organizations (i.e. NGOs), and international bodies (i.e. UN, IOM, COE) is key to developing inclusive and effective policies. Strategies to engage diverse stakeholders, for instance NGOs and other organizations from the civil societies, should be explored to ensure comprehensive perspectives in the policymaking process and avoiding double collection of data where data is already available.

In conclusion, to the present time gender-disaggregated data and gender mainstreaming in women’s migration policies across Europe reflect some challenges. Addressing methodological variations, improving policy implementation, recognizing intersectionality, strengthening legal frameworks, enhancing capacity, implementing effective monitoring, and fostering collaboration among stakeholders are critical steps forward. This analysis provides a foundation for future research and policy initiatives to advance gender equality in the context of women’s migration in the EU.

### VI. WEMov’s approach

COST Action network (WEMov) has been actively engaged in projects aimed at enhancing the visibility of migrant women across Europe. As part of its initiatives, WEMov has collected national, regional (EU) and international available and open-access datasets from European and COST member countries that specifically focus on women’s migration. We have also addressed the dearth of data on women’s migration through primary sources, which enable to showcase women’s migration across countries and centuries, thus helping comparative analysis ( https://www.womenonthemove.eu/primary-sources/#repository). Besides, we have included landmarks that mostly show women migrants’ artists or politician, which has demonstrated the presence of migrant women in Europe, despite only well-known, maybe not for their migrant status, elite women ( https://www.womenonthemove.eu/landmarks-exhibitions/). At the same time, we have created a timeline and a map with the most important migrant women events from the 16th century to nowadays, facing remarkable problems in demonstrating their existence and their specific migration patterns because most of the statistics were not available.

As a matter of fact, we have encountered challenges due to the absence of common methodologies of collection of data based on the definitions of migrant, the sex and the gender connotation as well as the time availability of some data compared to other, for instance data from the end of the cold war is more available, difficulties in data accessibility, and a lack of comprehensive data in certain countries.

In our commitment to knowledge exchange, we have organized cross-national seminars addressing various research topics and issues among COST members, students, and stakeholders. During these seminars, the critical importance of gender mainstreaming and the need for gender-disaggregated data have emerged as vital considerations.

As part of our multifaceted approach, we are in the process of creating a documentary that explores the diverse perceptions of migrant women in Europe, both from EU and non-EU countries. Through interviews, it has become evident that a prevailing focus on portraying women solely as vulnerable individuals migrating for family reasons or to reunite with male partners is impeding the development of more comprehensive policies. Some interviewees have highlighted the need for a shift towards policies that leverage networks of assistance and provide training support for women, facilitating their integration into new countries. Additionally, concerns have been raised about the limitations of humanitarian migration policies in addressing the broader needs of migrant women and advocate for more inclusive and effective policies for migrant women across Europe.

### VII. Challenges

Inconsistent Data Collection: The collection of gender-disaggregated data remains inconsistent across EU member states, hindering the development of effective policies.

Limited Gender Mainstreaming: While the concept of gender mainstreaming is well-established, its consistent implementation in migration policies is lacking.

Intersectional Considerations: Policies often overlook the intersectionality of factors affecting migrant women, leading to incomplete support and giving emphasis only on vulnerabilities instead of women’s agency and resources.

### VIII. Recommendations

Create a standardized Data Collection: The EU should establish standardized methodologies for collecting gender-disaggregated data in the context of migration and create a group of experts to monitor the collection, write reports and visit different countries to monitor collection practices on an annual basis.

Create an EU database based on a standardized data collection: This will foster the understanding of researchers and policymakers on a cross-country level. This database should be made available online and usable to all.

Strengthen Gender Mainstreaming: Member states must prioritize the integration of gender mainstreaming into all migration-related policies and programs. This commitment should be fortified by adherence to EU guidance, which can be effectively enforced through the establishment of a peer-review system involving three EU countries’ experts. The first country expert in this system would be responsible for crafting the initial policy or piece of legislation. The second country expert would assume the role of reviewing the policy, providing crucial insights and perspectives. Finally, the third country expert would engage in a debate role, overseeing the adherence to gender mainstreaming practices and ensuring their effective implementation. This structured peer-review process aims to enhance the quality and inclusivity of migration policies across the EU, fostering a comprehensive and gender-sensitive approach.

Evaluate gender-mainstreaming implementation at the national level: The European Union ought to establish a dedicated expert body comprising three members from each country, representing distinct perspectives: one from a top-down institution, such as a ministry; another from a non-governmental organization (NGO); and the third from the realm of researchers. This body, situated within the EU, would play a crucial role in monitoring the integration of gender considerations into migration policies across the member states.

## Data Availability

No data are associated with this article.
