# Supplementary material for: Gender mainstreaming and gender-disaggregated data on migrant women: WEMov’s Open Letter
Source: Open Res Eur. 2025 Jun 12;4:197. Originally published 2024 Sep 9. [Version 2] doi: 10.12688/openreseurope.18103.2 (PMC12264470; doi:10.12688/openreseurope.18103.2)
Supplement: Peer review history

# Reviewer report: Reviewer response for version 2

**DOI:** 10.21956/openreseurope.22036.r55433

**Published:** 2025-08-21

**Authors:** Hari KC

**Affiliations:** 1 Toronto Metropolitan University, Toronto, Ontario, Canada

 I am pleased to note that the authors have thoughtfully addressed the concerns I raised, particularly with respect to the conceptual distinction between sex- and gender-disaggregated data, the political dimensions of data categorization, and the importance of foregrounding the WEMov project’s contributions. The revised brief has enhanced clarity and depth.

Where applicable, are recommendations and next steps explained clearly for others to follow? (Please consider whether others in the research community would be able to implement guidelines or recommendations and/or constructively engage in the debate)

Yes

Does the article adequately reference differing views and opinions?

Partly

Are all factual statements correct, and are statements and arguments made adequately supported by citations?

Yes

Is the rationale for the Open Letter provided in sufficient detail? (Please consider whether existing challenges in the field are outlined clearly and whether the purpose of the letter is explained)

Yes

Is the Open Letter written in accessible language? (Please consider whether all subject-specific terms, concepts and abbreviations are explained)

Partly

Reviewer Expertise:

Gender and migration; global migration governance; labour migration in Asia

I confirm that I have read this submission and believe that I have an appropriate level of expertise to confirm that it is of an acceptable scientific standard.

# Reviewer report: Reviewer response for version 2

**DOI:** 10.21956/openreseurope.22036.r55435

**Published:** 2025-07-16

**Authors:** andrea spehar

**Affiliations:** 1 University of Gothenburg, Gothenburg, Västra Götaland County, Sweden

I would like to let you know that the revisions made to the article are appropriate. I have no objections to the current version and consider it ready to proceed.

Where applicable, are recommendations and next steps explained clearly for others to follow? (Please consider whether others in the research community would be able to implement guidelines or recommendations and/or constructively engage in the debate)

Yes

Does the article adequately reference differing views and opinions?

Partly

Are all factual statements correct, and are statements and arguments made adequately supported by citations?

Yes

Is the rationale for the Open Letter provided in sufficient detail? (Please consider whether existing challenges in the field are outlined clearly and whether the purpose of the letter is explained)

Yes

Is the Open Letter written in accessible language? (Please consider whether all subject-specific terms, concepts and abbreviations are explained)

Yes

Reviewer Expertise:

Comparative migration and migrant integration policy analysis

I confirm that I have read this submission and believe that I have an appropriate level of expertise to confirm that it is of an acceptable scientific standard.

# Reviewer report: Reviewer response for version 1

**DOI:** 10.21956/openreseurope.19568.r44467

**Published:** 2024-11-08

**Authors:** Hari KC

**Affiliations:** 1 Toronto Metropolitan University, Toronto, Ontario, Canada

The brief provides a critical assessment of the current efforts being undertaken by the EU member states pertaining to gender mainstreaming of migration policies and gender-disaggregated data on women migrants.

 It also highlight the key gaps and challenges that remain and are yet to be addressed when it comes to adopting and implementing a gender equality principle in migration policies and data and proposes some excellent recommendations to address the lacuna.

 Despite these strengths of the brief, here are some comments and suggestions that could help add to its clarity and methodological rigour.

 In the first place, though the authors state that there is a need of making a distinction between sex-disaggregated and gender-disaggregated data, there still seems to be some conflation and obfuscation of these terms. Related to this, the authors rightly stress on the need to collect and analyze gender-disaggregated data about women migrants but it is important to go beyond if we consider gender as a social-political construct and it relates to unequal power relations. What this means is that it is critically important to produce gender-disaggregated data (not mere collect based on gender biased or gender blind categories) by creating new categories -- the categories that can speak to and capture the gendered experiences and outcomes. The authors mention about such approaches they have taken in the Women on the Move project but this research which informs the brief needs to be foregrounded. Gender-disaggregation of migration data is not thus a mere technical issue but a deeply political one as discussed in this piece available at https://publications.iom.int/system/files/pdf/Gender-and-Migration-Data.pdf.

 It is stated that there are some gender-disaggregated data on women migrants collected by some international organizations (e.g., World Bank) - it would be helpful to explain how how those data are gender=disaggregated. Also, about the two terms discussed in the brief (gender mainstreaming and gender-disaggregated data) – it would help if some discussion is provided as to how these the two can/should inform one another highlighting the interplay between gender mainstreaming of migration policies and gender disaggregated data on women migrants. Lastly, it is true that conventional migration scholarship has largely overlooked the presence of women migrants (Boyd & Grieco (2003), but there has been a plethora of scholarship on gender and migration since 2003.

Where applicable, are recommendations and next steps explained clearly for others to follow? (Please consider whether others in the research community would be able to implement guidelines or recommendations and/or constructively engage in the debate)

Yes

Does the article adequately reference differing views and opinions?

Partly

Are all factual statements correct, and are statements and arguments made adequately supported by citations?

Yes

Is the rationale for the Open Letter provided in sufficient detail? (Please consider whether existing challenges in the field are outlined clearly and whether the purpose of the letter is explained)

Yes

Is the Open Letter written in accessible language? (Please consider whether all subject-specific terms, concepts and abbreviations are explained)

Partly

Reviewer Expertise:

Gender and migration; global migration governance; labour migration in Asia

I confirm that I have read this submission and believe that I have an appropriate level of expertise to confirm that it is of an acceptable scientific standard, however I have significant reservations, as outlined above.

# Response

**Published:** 2025-05-02

**Authors:** Marie-José Ruiz

**Affiliations:** Anglophone Studies, Universite de Picardie Jules Verne, Amiens, Hauts-de-France, France

We thank the reviewer for their generous appraisal of the policy brief and their constructive comments aimed at strengthening its clarity and conceptual depth. We are particularly grateful for the insights on terminology and the political dimensions of data collection.

**1. Clarifying the Distinction Between Sex- and Gender-Disaggregated Data** We agree that the distinction between **sex-disaggregated** (biological differences) and **gender-disaggregated** (socially constructed roles, expectations, and power relations) data must be clearly articulated. **Gender-disaggregated data must be generated in ways that capture lived experiences and social positioning**, not just binary or surface-level categorization. **Gendered data collection is not merely technical but political**, reflecting whose experiences are made visible or invisible.

**2. Foregrounding WEMov’s Contribution and the Political Nature of Data Categories** We appreciate the reviewer’s suggestion to foreground the **research conducted within the COST Action Women on the Move (WEMov)**. We highlight our efforts to uncover under-documented aspects of women’s migration through historical and qualitative data ; the **challenges we faced in sourcing intersectional and gender-aware data **; how WEMov’s work contributes to **challenging gender-blind or biased categories** in mainstream migration data systems. We have **created new, meaningful categories** that reflect the gendered and intersectional realities of migrants, which is essential. We state explicitly that **data production itself is shaped by power relations**, and thus, gender-disaggregation must be part of a transformative agenda, not only an analytical tool.

**3. Expanding on How Existing International Data Sets Are Gender-Disaggregated** We thank the reviewer for encouraging greater specificity. We have elaborated on how gender is (or is not) represented in the data produced clarifying where gender variables exist, what they actually measure ; where gaps persist (e.g., lack of data on motives for migration, reproductive health, or remittances by gender) ; the limitations of these data sets in capturing **nuanced, intersectional gender dynamics**.

**4. Interplay Between Gender Mainstreaming and Gender-Disaggregated Data** We fully agree with the importance of articulating the **interdependence between gender-disaggregated data and gender mainstreaming**. **Gender-disaggregated data is foundational to gender mainstreaming** as it allows for evidence-based design, implementation, and evaluation of gender-sensitive policies. **Gender mainstreaming**, in turn, can **drive better data collection** by pushing institutions to recognize whose experiences need to be made visible in policymaking.

**5. Acknowledging Broader Gender and Migration Scholarship** We appreciate the reviewer’s important reminder that gender and migration scholarship has grown substantially since the early 2000s. **Additional Edits**

- All technical terms and abbreviations are **accessible to a broad audience**.
- The recommendations section reflects the **interplay of data, ethics, and policy** and explicitly notes the **need for gender-aware data frameworks** that go beyond binaries or administrative convenience.

We sincerely thank the reviewer for highlighting these important areas in the brief’s argument and practical value.

**Stellamarina Donato & Marie Ruiz**

# Reviewer report: Reviewer response for version 1

**DOI:** 10.21956/openreseurope.19568.r44461

**Published:** 2024-10-24

**Authors:** andrea spehar

**Affiliations:** 1 University of Gothenburg, Gothenburg, Västra Götaland County, Sweden

The open letter makes a significant contribution by positing that gender mainstreaming and the utilisation of gender-disaggregated data are pivotal tools for addressing gender disparities and ensuring evidence-based policymaking in relation to international migration. It elucidates the policy implications of missing or incomplete gender-disaggregated data in relation to international migration.

 I would encourage the authors to address additional aspects to reinforce their core arguments and policy recommendations.

 The article presents a compelling argument for the necessity of disaggregated gender data in order to shed light on the existing inequalities faced by women and girls in the context of migration processes. It is, however, important to emphasise that the concept of gender is not to be confused with the term 'women'. The term 'gender' encompasses women, men and other gender groups, and the unequal relations between them. Discussions on gender frequently concentrate on women, as they are the group most affected by gender inequality.  While there is a substantial body of research on migration, return and reintegration that focuses on women, there is a paucity of research examining the experiences of LGBTQI+ people. The disaggregation of data by gender should facilitate the identification of specific vulnerabilities and needs, thereby enhancing the design, implementation and evaluation of reintegration support programmes for returnees of all genders and intersecting identities based on race, religion, caste, colour, etc. This also necessitates the reconsideration of sampling frames and methodological designs, in addition to the expansion of variables and indicators to more accurately assess the lived experiences of migrants of all genders.

 The open letter would be enhanced by the inclusion of a discussion of the challenges associated with transparency, accountability and ethics in the context of migration data. It can be reasonably argued that a dearth of sex- or gender-disaggregated migration data may have the effect of undermining the engagement of civil society organisations (CSOs) and the provision of services for migrants. Concurrently, there are ethical challenges inherent to the availability of such data. The provision of sensitive information pertaining to gender and international migration can exacerbate the precarious and vulnerable circumstances of certain groups. To illustrate, the collection and dissemination of data pertaining to sexual and reproductive health among migrant workers (such as pregnancy test results) on tied work permits can exacerbate the precarious situation of women migrant workers in the event that the confidentiality of such information is not guaranteed. Furthermore, the sharing of data on diverse gender identities and sexual orientations with officials in certain contexts may result in migrants being subjected to discriminatory practices, abuse, detention, or deportation.

Where applicable, are recommendations and next steps explained clearly for others to follow? (Please consider whether others in the research community would be able to implement guidelines or recommendations and/or constructively engage in the debate)

Yes

Does the article adequately reference differing views and opinions?

Partly

Are all factual statements correct, and are statements and arguments made adequately supported by citations?

Yes

Is the rationale for the Open Letter provided in sufficient detail? (Please consider whether existing challenges in the field are outlined clearly and whether the purpose of the letter is explained)

Yes

Is the Open Letter written in accessible language? (Please consider whether all subject-specific terms, concepts and abbreviations are explained)

Yes

Reviewer Expertise:

Comparative migration and migrant integration policy analysis

I confirm that I have read this submission and believe that I have an appropriate level of expertise to confirm that it is of an acceptable scientific standard, however I have significant reservations, as outlined above.

# Response

**Published:** 2025-05-02

**Authors:** Marie-José Ruiz

**Affiliations:** Anglophone Studies, Universite de Picardie Jules Verne, Amiens, Hauts-de-France, France

We appreciate the reviewer’s thoughtful and constructive feedback, which has provided valuable insight for enhancing the policy brief. We are grateful for the recognition of the importance of gender mainstreaming and gender-disaggregated data in the context of migration policy. **1. Clarifying the Concept of Gender** We acknowledge the reviewer’s point that the term “gender” should not be conflated with “women” alone, as gender encompasses women, men, and other gender identities, as well as the unequal relations between them. **Gender** is considered as a broader social construct and **women** as a specific group most affected by gender inequality. A **discussion of LGBTQI+ migrants** emphasizes the need for intersectional approaches in both **data collection** and **policy design**, and highlights the **specific vulnerabilities** and needs of LGBTQI+ migrants, as well as the **importance of disaggregated data** in designing inclusive and equitable reintegration programs for all genders. We suggests **reconsidering sampling frames** and **methodological designs** to ensure the inclusion of a broader spectrum of gender identities and intersecting factors such as **race, religion, caste, and color**. **2. Transparency, Accountability, and Ethics in Migration Data** We greatly appreciate the reviewer’s point about the **ethical challenges** surrounding the collection and dissemination of sensitive migration data. We discuss how the **confidentiality of sensitive data** (e.g., sexual and reproductive health, gender identity) is critical to avoid exposing migrants to further **vulnerability, abuse, or discrimination**. This is necessary for **ethical safeguards** when collecting, sharing, and disseminating data. Specifically, we explore how data on **gender and sexual orientation** should be handled with the utmost care to prevent harm. We have included **examples** of how the sharing of such data, especially in **work-permit contexts**, could result in **discriminatory practices** or **detention** and **deportation** in certain environments. We also stress the need for **transparent data practices** that guarantee **privacy and security** for vulnerable populations. **3. Expanding the Policy Recommendations** There is a need for strengthened policies around **data disaggregation**, the inclusion of **LGBTQI+ migrants**, and the **ethical challenges** associated with gender-sensitive data collection. We address the importance of including **LGBTQI+ migrants** in data collection and policy design ; the necessity of using **intersectional data** to better understand the **complexities** faced by migrants of different genders and backgrounds ; the critical need to establish **ethical standards** for the collection and sharing of sensitive migration data, including data on sexual and reproductive health and gender identities, with a clear emphasis on safeguarding **privacy** and **confidentiality**. **Additional Adjustments** **There are differing views and ppinions**, particularly in relation to how **gender-disaggregated data** intersects with other factors like **race**, **ethnicity**, and **sexual orientation**. All **subject-specific concepts** are well-explained and accessible to a wider audience. We thank the reviewer again for their invaluable feedback and look forward to the continued development of this work. **Stellamarina Donato & Marie Ruiz**

# Reviewer report: Reviewer response for version 1

**DOI:** 10.21956/openreseurope.19568.r44463

**Published:** 2024-10-17

**Authors:** Maria Caterina La Barbera

**Affiliations:** 1 Consejo Superior de Investigaciones Científicas, Madrid, Spain

The open letter addresses an important topic, is well written, and makes concrete recommendations.

 Two points need to be revised and addressed first.

 1. The article makes a good point when arguing that gender disaggregated data is needed. However, gender does not put all migrant women in the same situation. Gender interacts with ethnicity, education, employment, sexual orientation, nationality of origin and administrative situation (type of residence permit, irregular situation, etc.). An intersectional perspective is also needed when arguing for disaggregated data. In other words, we need data that allows us to take into account gender, motherhood, ethnicity, education, employment, sexual orientation, nationality of origin and administrative situation. Intersectionality could be introduced from the start when talking about granularity ("different aspects such as employment, health care, education and their contribution to the local economy").

 2. A critical reflection on the use of disaggregated data is needed. Disaggregated data needs to be complemented by qualitative studies that allow to understand the lived impact of structural inequalities beyond statistics. On the unavailability and misuse of disaggregated data, see La Barbera M, Espinosa-Fajardo J, Caravantes P. Implementing Intersectionality in Public Policies: Key factors in the City of Madrid, Spain. Politics & Gender. 2023;19(3):675-702. doi:10.1017/S1743923X22000241

Where applicable, are recommendations and next steps explained clearly for others to follow? (Please consider whether others in the research community would be able to implement guidelines or recommendations and/or constructively engage in the debate)

Yes

Does the article adequately reference differing views and opinions?

Partly

Are all factual statements correct, and are statements and arguments made adequately supported by citations?

Yes

Is the rationale for the Open Letter provided in sufficient detail? (Please consider whether existing challenges in the field are outlined clearly and whether the purpose of the letter is explained)

Yes

Is the Open Letter written in accessible language? (Please consider whether all subject-specific terms, concepts and abbreviations are explained)

Yes

Reviewer Expertise:

Intersectionality, gender discrimination, human rights, European Union, policy implementation

I confirm that I have read this submission and believe that I have an appropriate level of expertise to confirm that it is of an acceptable scientific standard, however I have significant reservations, as outlined above.

## References

[1] : Implementing Intersectionality in Public Policies: Key Factors in the Madrid City Council, Spain. *Politics & Gender* .2023;19(3) : 10.1017/S1743923X22000241 675-702 10.1017/S1743923X22000241

# Response

**Published:** 2025-05-02

**Authors:** Marie-José Ruiz

**Affiliations:** Anglophone Studies, Universite de Picardie Jules Verne, Amiens, Hauts-de-France, France

We thank the reviewer for their thoughtful and generous assessment of the policy brief, and for highlighting further developments.

**1. Incorporating an Intersectional Perspective into Disaggregated Data**

 We appreciate the reviewer’s observation that gender alone does not capture the full scope of migrant women’s diverse experiences. We agree for generalised need for **intersectional data** outlining challenges in data collection. We emphasize that gender-disaggregated data must go beyond binary distinctions and integrate variables such as **ethnicity, education level, employment status, sexual orientation, nationality of origin, motherhood, and administrative status**. These dimensions interact to shape women migrants’ vulnerabilities and agency, and they are crucial for producing more meaningful analyses and effective policy responses.

**2. Critical Reflection on the Use of Disaggregated Data**

 We acknowledge the need to critically assess how disaggregated data is used and to underscore the limitations of statistical data alone to reflect on the potential misuse or overreliance on quantitative data in migration policy. The **complementary role of qualitative research** in revealing the lived experiences of structural inequalities cannot be fully captured by numerical indicators. We believe this brief ensures that gender-disaggregated data is not framed as a neutral or purely technical tool, but as a political and epistemological instrument that must be handled with care and accountability. We are grateful that the reviewer confirmed the clarity of the rationale, the factual accuracy of the statements, and the accessibility of the language used. Once again, we extend our sincere thanks to the reviewer for their important suggestions. **Marie Ruiz & Stellamarina Donato **COST Action CA19112 – Women on the Move **Andrea** **Spehar**, University of Gothenburg, Gothenburg, Västra Götaland County, Sweden We appreciate the reviewer’s thoughtful and constructive feedback, which has provided valuable insight for enhancing the policy brief. We are grateful for the recognition of the importance of gender mainstreaming and gender-disaggregated data in the context of migration policy.

**1. Clarifying the Concept of Gender**

 We acknowledge the reviewer’s point that the term “gender” should not be conflated with “women” alone, as gender encompasses women, men, and other gender identities, as well as the unequal relations between them. **Gender** is considered as a broader social construct and **women** as a specific group most affected by gender inequality. A **discussion of LGBTQI+ migrants** emphasizes the need for intersectional approaches in both **data collection** and **policy design**, and highlights the **specific vulnerabilities** and needs of LGBTQI+ migrants, as well as the **importance of disaggregated data** in designing inclusive and equitable reintegration programs for all genders. We suggests **reconsidering sampling frames** and **methodological designs** to ensure the inclusion of a broader spectrum of gender identities and intersecting factors such as **race, religion, caste, and color**.

**2. Transparency, Accountability, and Ethics in Migration Data**

 We greatly appreciate the reviewer’s point about the **ethical challenges** surrounding the collection and dissemination of sensitive migration data. We discuss how the **confidentiality of sensitive data** (e.g., sexual and reproductive health, gender identity) is critical to avoid exposing migrants to further **vulnerability, abuse, or discrimination**. This is necessary for **ethical safeguards** when collecting, sharing, and disseminating data. Specifically, we explore how data on **gender and sexual orientation** should be handled with the utmost care to prevent harm. We have included **examples** of how the sharing of such data, especially in **work-permit contexts**, could result in **discriminatory practices** or **detention** and **deportation** in certain environments. We also stress the need for **transparent data practices** that guarantee **privacy and security** for vulnerable populations.

**3. Expanding the Policy Recommendations**

 There is a need for strengthened policies around **data disaggregation**, the inclusion of **LGBTQI+ migrants**, and the **ethical challenges** associated with gender-sensitive data collection. We address the importance of including **LGBTQI+ migrants** in data collection and policy design ; the necessity of using **intersectional data** to better understand the **complexities** faced by migrants of different genders and backgrounds ; the critical need to establish **ethical standards** for the collection and sharing of sensitive migration data, including data on sexual and reproductive health and gender identities, with a clear emphasis on safeguarding **privacy** and **confidentiality**. **Additional Adjustments** **There are differing views and ppinions**, particularly in relation to how **gender-disaggregated data** intersects with other factors like **race**, **ethnicity**, and **sexual orientation**. All **subject-specific concepts** are well-explained and accessible to a wider audience. We thank the reviewer again for their invaluable feedback and look forward to the continued development of this work.

**Stellamarina Donato & Marie Ruiz**
